# iPSCs-based generation of vascular cells: reprogramming approaches and applications

**DOI:** 10.1007/s00018-017-2730-7

**Published:** 2017-12-14

**Authors:** Diana Klein

**Affiliations:** Institute for Cell Biology (Cancer Research), University Hospital Essen, University of Duisburg-Essen, Virchowstr. 173, 45122 Essen, Germany

**Keywords:** iPSC, Reprogramming, Differentiation, Vascular cell, Endothelial cell, Smooth muscle cell

## Abstract

Recent advances in the field of induced pluripotent stem cells (iPSCs) research have opened a new avenue for stem cell-based generation of vascular cells. Based on their growth and differentiation potential, human iPSCs constitute a well-characterized, generally unlimited cell source for the mass generation of lineage- and patient-specific vascular cells without any ethical concerns. Human iPSCs-derived vascular cells are perfectly suited for vascular disease modeling studies because patient-derived iPSCs possess the disease-causing mutation, which might be decisive for full expression of the disease phenotype. The application of vascular cells for autologous cell replacement therapy or vascular engineering derived from immune-compatible iPSCs possesses huge clinical potential, but the large-scale production of vascular-specific lineages for regenerative cell therapies depends on well-defined, highly reproducible culture and differentiation conditions. This review will focus on the different strategies to derive vascular cells from human iPSCs and their applications in regenerative therapy, disease modeling and drug discovery approaches.

## Introduction

Induced pluripotent stem cells (iPSCs) are reprogrammed cells that have features similar to embryonic stem cells, such as self-regeneration without restriction and differentiation into different tissue or cell types, including vascular cells [1]. Connected with these cells is the hope that 1 day they could be used as an inexhaustible source to restore damaged or diseased body tissue [2, 3].

Artificial reprogramming of somatic cells produces cells that maintain their great potential for medical use but research is associated with less ethical problems than embryonic stem cells resulting from the internal cell mass of blastocysts (embryoblasts) [4, 5]. In principle, iPSCs have the potential to differentiate into various adult human cell types and tissues. Although it is a long way from a few cells in a Petri dish to a complex, functional tissue that could replace an injured or diseased organ, the exciting and medically important duty of iPSCs research is to understand the basic controlling mechanisms of nature and to learn how stem cells approach differentiated cells and transform into certain tissues. Furthermore, iPSCs turned out to be a realistic method for obtaining patient-specific stem cells and using them for the investigation of diseases and their therapy, e.g. development of drugs or toxicological testing of chemical substances, as well as their use as target cells for individualized and targeted gene therapy [6].

The vascular system is essential for embryonic development and adult life and it is the first organ system to develop during vertebrate embryogenesis [7, 8]. Herein, endothelial cells arise from mesodermal precursors around embryonic day E8.5, and the first primitive vascular network remodels and matures between E9.5 and E12.5 [7, 9–11]. These remodeling and maturation processes involve the recruitment of vascular mural cells: pericytes ensheath capillary endothelial cells, whereas smooth muscle cells (SMC) surround endothelial cells in arteries, arterioles, venules and veins, which is critical for proper vascular development, stabilization, and maintenance. An ordered remodeling is an absolute prerequisite to preserve the sensitive relationship between resilience and stability of the vessels [12]. In the healthy state adult blood vessels are usually quiescent, which means a non-proliferating, anti-thrombotic, anti-inflammatory and non-angiogenic default status of resting endothelial cells. SMC in the arterial wall are mostly quiescent but can display a contractile phenotype in adults. Under pathophysiological conditions, vascular remodeling after endothelial dysfunction or damage is directly or indirectly associated with multiple common human diseases: atherosclerosis, thrombosis, hypertension, ischemic diseases, congenital vascular lesions (aneurysms, fibromuscular hyperplasia, and stenosis in collaterals), shear stress, irradiation, and even tumor growth [13, 14].

The task of blood vessels involves transporting of all the nutrients as well as of their metabolites, e.g. of toxic waste. The goal is an optimal supply of all cells of the body and the blood circulation with vital substances [15–17]. Anatomically, the wall of all larger blood vessels—whether arteries or veins—viewed from the inside outwards is basically constructed from three different layers. The innermost layer (also called tunica intima), which is composed of a single continuous layer of endothelial cells, is in direct contact with the blood flow and mediates the exchange of nutrients and cells with the circulation. The middle layer (tunica media), which consists of SMC, is responsible for the maintenance of the vessel tone and elasticity. The outer layer (adventitia), which consists of connective tissue, harbors fibroblasts and the small network of vessels that provides oxygen and nutrients to the cells in the vessel wall (vasa vasorum) [18]. Herein, the endothelial cells are key components for maintaining the function of the vessels by regulating endothelial permeability, modulating the vascular tone and regulating blood coagulation. The vessel diameter can be changed by the contraction of their muscle cell layer(s), whereby blood vessels control the distribution of blood volume and thus regulate the oxygen supply. In addition, it keeps individual body regions in the core temperature.

Vascular damage and dysfunction are implicated in the development of vascular diseases and cardiovascular pathological changes. Therefore, the therapeutically use of autologous cells for tissue regeneration is a ray of hope, which includes both local transplantation of the vascular cells to injured organs and the engineering of organs. Cell replacement therapy with vascular cells and vascular bypass grafts with autologous blood vessels or synthetic vascular grafts are the main treatments for certain cardiovascular diseases, such as coronary heart disease, aortic aneurysm, dissection, and peripheral vascular disease [19–23].

**Fig. 1:**
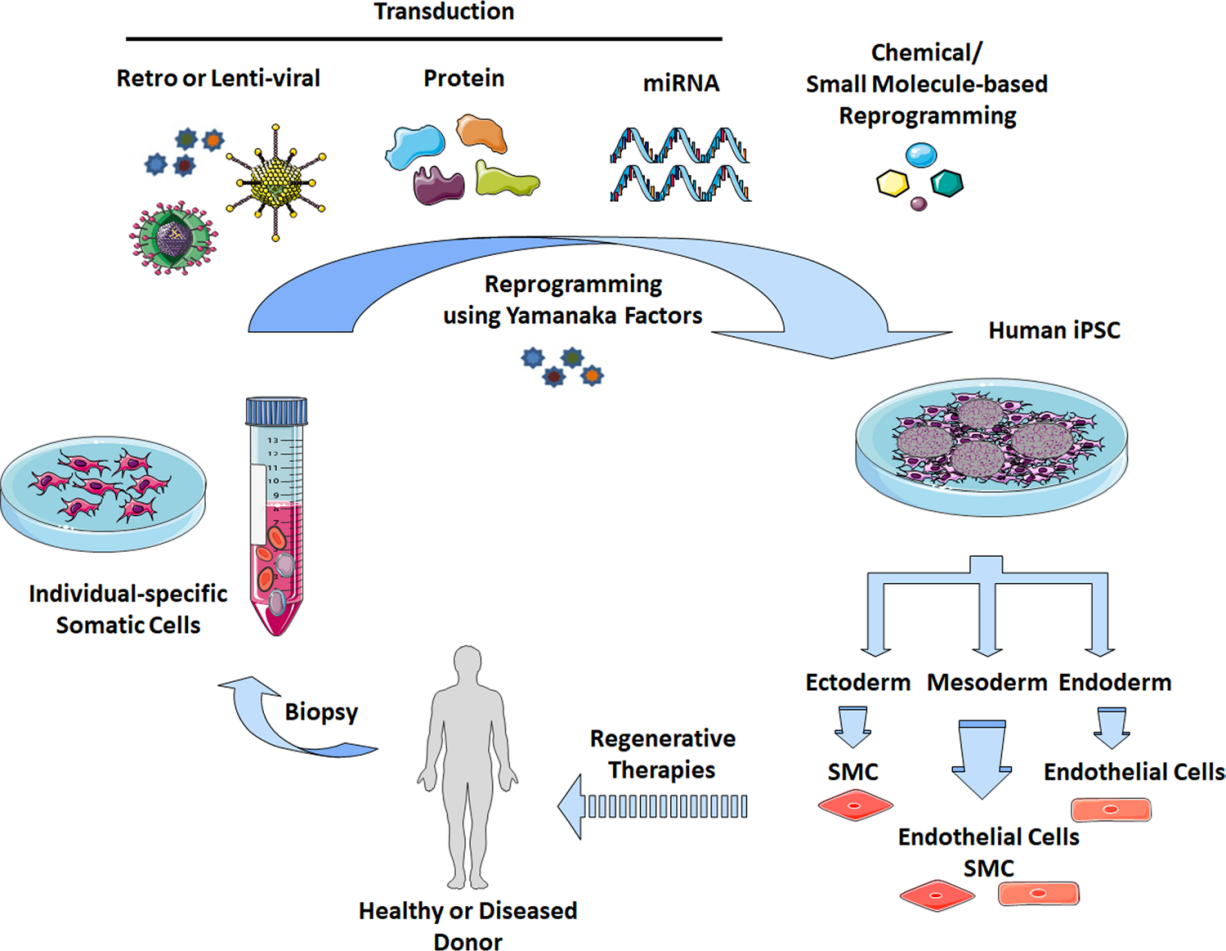
Induced reprogramming of somatic cells. Somatic cells (e.g. fibroblasts, peripheral blood cells) can be isolated from individual healthy or diseased donors (biopsy) and directly reprogrammed into human iPSCs by the introduction of the common transcription factors *OCT4*, *SOX2*, *KLF4* and *c*-*MYC* (Yamanaka factors) via retro-/lentiviral transduction, protein and microRNA transduction, or by chemical/small molecule-based reprogramming strategies. iPSCs were characterized by indefinite self-renewal and pluripotent differentiation capacities, and thus represent an attractive source to generate unlimited cell numbers for targeted differentiation, in principle, into the entire range of cell types found in the body via multiple lineages (ectoderm, endoderm and mesoderm). The generation of patient- and disease-specific iPSCs is a valuable tool for (1) regenerative therapies, e.g. restoration of function through transplantation of manufactured cells and tissues, (2) exploring disease etiology and associated pathophysiologic mechanisms, and (3) developing novel drugs and toxicology screening. iPSC, induced pluripotent stem cell; SMC, smooth muscle cell; miRNA, microRNA

The main limitations for the use of vascular cells for the treatment of cardiovascular diseases were the sources and amount of the cells. In general, their proliferative potential decreases with increasing donor age and intensive ex vivo expansion can limit their properties such as long term plasticity and result in a functional decline. In addition, adult cells may contain more DNA abnormalities (caused by sunlight, toxins and errors in making DNA copies) during the course of a lifetime [24–26]. Thus, finding a reliable source of cells remains an important problem. To circumvent many of these issues, an alternative method to gain vascular cells is the in vitro differentiation of iPSCs, which are one of the most appropriate basic cell sources (Fig. 1) [27–30]. Human iPSC-derived vascular cells display similar features with mature vascular cells at the genetic and functional levels.

The unlimited proliferation potential of iPSCs and their capability to differentiation into virtually every cell type in the human body is of great significance to explore alternative cell sources capable of generating functional endothelial cells and SMC. Furthermore, the generation of structures to repair damaged endothelium for vascular regeneration as well as blood vessels en bloc were desired because endothelial cell regeneration is a slow and insufficient process [31, 32]. Tissue-engineered vascular grafts for examples are promising novel alternatives to replace diseased vessels. Herein generating enough functional and clinically usable vascular cells for conducting these vascular grafts remains a major challenge [21].

Beside the abundant origins of iPSCs the potential to generate patient individualized vascular cells that bypass the immunogenicity and ethical issues are central advantages of using iPSCs as vascular cell source. However, a possible therapeutic use of pluripotent stem cells still holds medical risks, namely the potential to generate teratomas. Therefore, only donor cells that have reached a particular differentiation stage could be used, which means that the iPSCs must first be brought to an ordered differentiation path. Thus, a major obstacle for using human iPSCs for therapy or to model disease remains the lack of reliable, efficient and scalable protocols to differentiate functionally mature adult cell types.

Based on progress in the research field, the present review aims to summarize the strategies and mechanisms of generating vascular cells through differentiating from human iPSCs, and to examine what this means for the potential application of cell therapy in the clinics.

## Reprogramming approaches

**Fig. 2:**
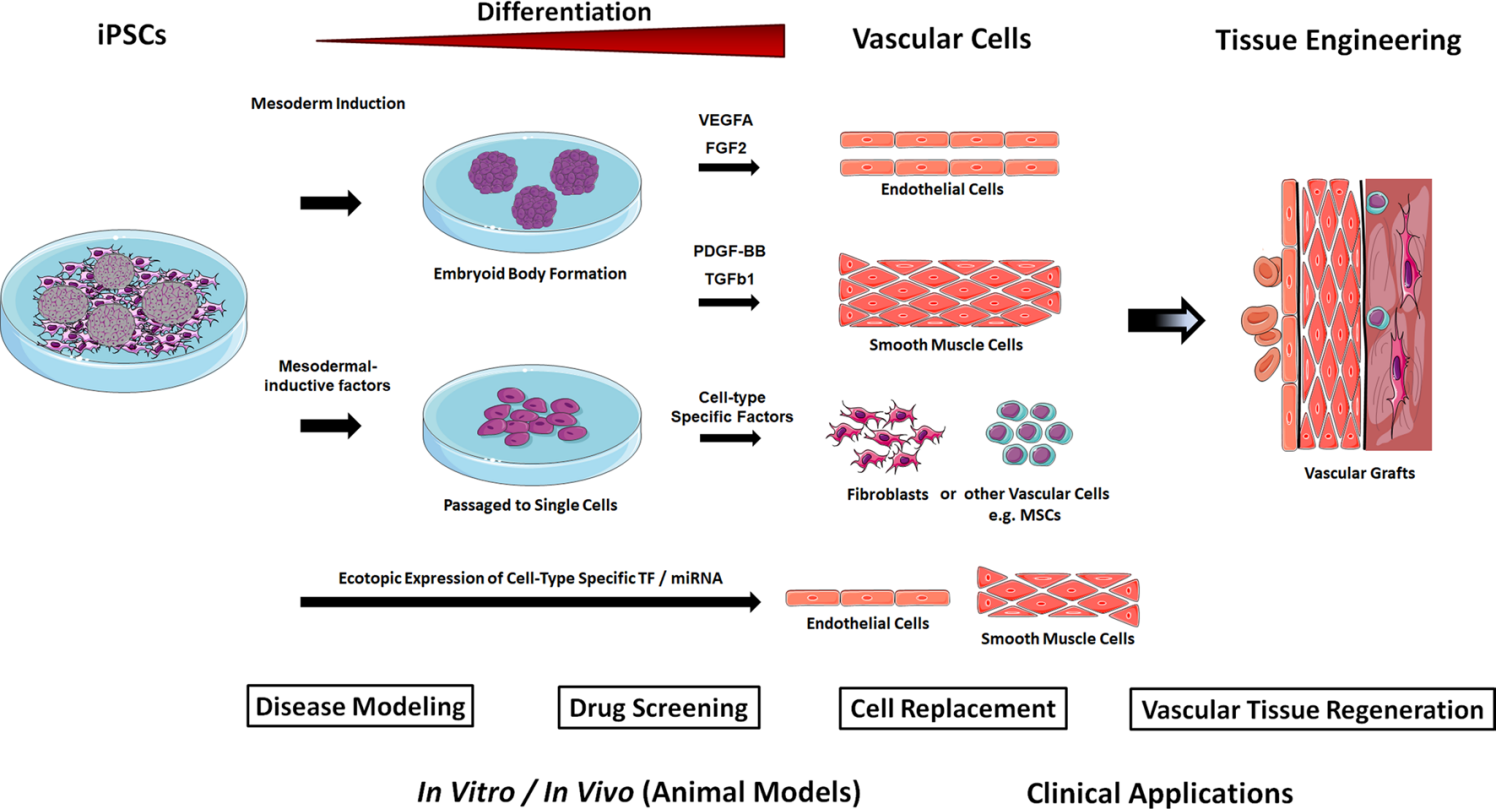
iPSCs-based generation of vascular cells. iPSCs are capable of self-renewal and differentiation into any cell type in the human body, and thus are attractive resources to generate unlimited numbers of vascular cells. Differentiation of iPSC is initiated by induction of mesodermal differentiation either in conditions that promote self-aggregation of the iPSCs into three-dimensional embryoid bodies (EB) with or without additional mesodermal-inductive factor treatment; or by the addition of mesodermal-inductive factors (BMP4, Activin A/Nodal, FGF2, and GSK3 inhibitors or WNT ligands) in chemically defined monolayer systems. Successive treatment with cell-type-specific growth factors for the desired cell types allows then the isolation and expansion of the selected vascular cells under chemically defined cell-culture conditions. Sorting for cell-type-specific cell surface markers using flow cytometry or immunomagnetic separation might further be used to increase purity of generated vascular cells. Human iPSC-derived vascular cells, in particular endothelial cells and smooth muscle cells turned out to be a realistic method for obtaining patient-specific cells and using them for the investigation of diseases and their therapy. These cells represent a potentially valuable tool for the development of robust and reproducible vascular tissues (stem cell-based vascular engineering) for disease modeling and drug screening applications. Hypothetically, vascular cells could also be obtained by a direct programming approach, namely by ectopic (over-)expression of vascular cell-specific transcription factors (TF) in human iPSCs or by the introduction of cell-type specific microRNA (miR) molecules that functions in RNA silencing and post-transcriptional regulation of vascular gene expression

In mammalian development, vascular progenitors mainly emerge from the lateral and posterior mesoderm [33]. Thus, vascular cells can be derived from differentiating iPSCs via three primary strategies: (1) iPSC differentiation towards the mesoderm followed by cell-type specific growth factor treatment, (2) culture on polymer coatings (extracellular matrix) in the presence of soluble, signaling molecules, and (3) genetic manipulation of iPSCs by ectopic expression of lineage or cell-type specific transcription factors (Fig. 2).

Induction of mesodermal differentiation can be achieved using conditions that promote self-aggregation of the iPSCs into three dimensional embryoid bodies (EB) or by the addition of mesoderm-inductive factors in chemically defined monolayer systems. The evolutionarily most ancient family members Nodal, Activin and BMP are members of the transforming growth factor β (TGFβ) superfamily of morphogens, which includes TGFβs, inhibins, bone morphogenetic proteins (BMPs), growth and differentiation factors (GDF), and others [34–36]. Combined gradients of Nodal and BMP signaling within the primitive streak initiate germ layer formation, control endoderm and mesoderm germ layer specification and also their subsequent patterning whilst blocking neuroectoderm formation [37, 38]. Activin/Nodal signaling achieves mesendoderm specification by interacting with other key signaling pathways, especially BMP and WNT, whereby WNT signaling also plays an essential function in mesoderm formation by blocking the PI3K/ERK pathway, and thereby inhibiting GSK3β, which is known to induce mesoderm differentiation [39, 40]. Another important factor is the fibroblast growth factor (FGF). FGF contributes to this process not only by promoting mesoderm formation, but also by inhibiting endodermal development [41]. Therefore, Activin and Nodal are essential for mesodermal induction, while FGF and WNT are in charge of its maintenance, and BMP is responsible for its patterning. Activin A/Nodal, BMP4, FGF2, and WNT ligands (e.g. WNT3A) or GSK3 inhibitors (canonical WNT activators, e.g. CHIR-99021) were the currently the most used mesoderm-inductive factors. Induced mesodermal progenitor cells were demonstrated to bear then a bi-potent differentiation potential with the ability to generate endothelial and SMC lineages [42].

No matter which way of mesodermal induction is chosen, the different lineages of vascular cells emerge then from the proliferating and differentiating iPSC in the presence of growth factors, extracellular matrix molecules, growth factor inhibitors, small molecules, and neutralizing antibodies that can be used to promote enrichment of the endothelial cells or SMC or the lineage of choice. To identify the cell lineage of choice and to enrich the cells using some form of cell sorting and isolation (flow cytometry sorting or immunomagnetic separation), monoclonal antibodies to identify specific cell surface molecules or genetic tagging of the iPSC with lineage-specific fluorescent reporter systems are required. Subsequent additions of numerous growth factors (as described below) further promote vascular cell proliferation, differentiation and eventual maturation during the in vitro culture.

## Reprogramming to endothelial cells

Endothelial cells originate mainly from mesodermal progenitors [43]. Shortly after gastrulation endothelial cells become organized into the primary vascular plexus which marks the onset of vascular development. Subsequent remodeling of the endothelial vasculature takes place, recruitment and integration of SMC and pericytes finally result in a complex vascular system. Thus, endothelial cells as the inner lining of blood vessels are diversified from simple capillaries formed by single endothelial cells into large multi-layered conduit vessels in the arterial, venous, and lymphatic systems. Beside vessel heterogeneity, even tissue heterogeneity of endothelial cells within specific tissues and organs prevail as revealed by specific angiocrine factors in modulating tissue functions [44–46]. Therefore, targeted profiles for tissue specific endothelial cell phenotypes have become of interest for scientists generating EC from iPSC [44, 47, 48].

Various methods have already been disclosed to generate endothelial cells from human iPSCs [44, 47, 49–52]. Two groups reported some of the first approaches to differentiate iPSCs toward endothelial cells in 2009. Taura et al. co-cultured human iPSCs with OP9 feeder cells for 10 days and observed the emergence of a VEGFR2-positive population with an endothelial cell differentiation capacity [53]. Using a similar approach, Choi et al. co-cultured different human iPSC lines with the same feeder cells for 8 days and then selected CD34- and CD31-double positive cell populations which could give rise to functional endothelial cells after additional 7 days under endothelial-promoting culture conditions [54].

Later on, more commonly endothelial lineage-committed cells could also be derived from EB formed by iPSCs. The first reported EB differentiation-based methods used high VEGF concentrations and resulted in less than 10% of CD31/PECAM1-positive cells after 2 weeks of culture [55–58]. Although recent advances have achieved higher efficiencies (up to 50% CD31-positive cells), the efficiencies are difficult to scale up because their methodology is based on EB/aggregate formation; and these results could not be replicated on several pluripotent stem cell lines [47, 59, 60]. Moreover, EB formation results in differentiation of iPSCs into various cell types, including endothelial cells, albeit inefficiently. EB differentiation is often time-consuming, with the peak expression of endothelial genes occurring after 10–15 days.

Nowadays, monolayer differentiation methods of feeder-free culture systems without the need of previous EB generation but with the combination of different culture substrates and chemical-defined conditions have been successfully applied to induce endothelial cells from iPSCs and were most commonly used. These improved methods resulted in an increase in differentiation fidelities, efficiencies and kinetics.

An alternative, technically more straight forward monolayer differentiation method was described in 2012 by Lippmann et al., who investigated a custom 2D human iPSC differentiation strategy that promotes neural and blood–brain-barrier endothelial cell co-differentiation [61]. Herein, human iPSCs (IMR90-4 cell line induced from fetal fibroblasts; DF6-9-9T and DF19-9-11T cell lines induced from foreskin fibroblasts) were maintained on irradiated mouse embryonic fibroblasts in standard unconditioned medium (DMEM/Ham’s F12 containing knockout serum replacer (KOSR), nonessential amino acids, l-glutamine, beta-mercaptoethanol, and human FGF2) [62, 63]. Shortly, bevor induction of endothelial differentiation iPSCs was passaged onto Matrigel in mTeSR1 medium. Differentiation was induced by switching to unconditioned medium lacking FGF2 (UM media). After 5–7 days when major morphological changes were observed the medium was replaced by an endothelial cell medium (human endothelial serum-free medium supplemented with FGF2 and platelet-poor plasma derived bovine serum). After additional 1–2 days differentiated iPSCs were plated onto polystyrene culture plates coated with a mixture of collagen-IV and fibronectin. This procedure yielded up to 95% combined neural and endothelial cell populations of the total differentiating culture at day 6 of UM treatment [61]. The 2 days-culture in endothelial cell medium further expanded the brain microvascular endothelial cells percentage (of GLUT1 and PECAM1/CD31-positive cells) to 66% in differentiating iPSC cultures. A slightly modification of the protocol mainly concerning the incubation times and not the factors used for differentiation of brain microvascular endothelial cells from iPSCs (BC1 cell line induced from blood cells) was recently provided by Katt et al. [64]. The addition of retinoic acid in differentiated endothelial cell cultures further upregulated expression levels of the mature endothelial cell marker VE-Cadherin and resulted in a significant increase in trans-endothelial electrical resistance to physiological values [64, 65].

In 2014, Orlova et al. described an elegant and very detailed protocol for the generation, expansion and functional analysis of endothelial cells (as well as pericytes) from human iPSCs under defined conditions [52]. Herein, substantial numbers of endothelial cells could be derived in only 2–3 weeks, including differentiation (10 days), immunomagnetic isolation (1 day), and expansion of CD31-positive endothelial cells (4–10 days) [52]. The differentiation protocol was tested and simplified for the use on multiple human iPSC lines, derived from either skin fibroblasts or blood outgrowth endothelial cells by transduction with either retroviruses or lentiviruses [52, 66]. Initiation of differentiation was performed in iPSC monolayers classically cultured on Matrigel-coated plates in mTeSR1 medium by replacing mTeSR1 medium with mesoderm induction medium [B(P)EL medium [57] containing Activin A, BMP4, VEGF and CHIR-99021 (small-molecule inhibitor of glycogen synthase kinase-3β)] for 2 days. On day 3, when first mesenchymal cells could be observed, mesoderm-inductive factors were replaced with vascular specification medium supplemented with VEGF and the TGFβ pathway small-molecule inhibitor SB431542 to support the expansion of endothelial cells while inhibiting the anti-proliferative activities of cell-derived TGFβ-like factors that are also present in the culture [52]. The earliest endothelial cells could be observed on day 6–7 of differentiation. At day 10 of differentiation, the total cell yield of mature VE-Cadherin and CD31-positive endothelial cells was about 10%. CD31-positive cells were further purified via immunomagnetic separation and expanded in endothelial cell serum-free medium (EC-SFM) to which platelet-poor plasma serum, VEGF and FGF2 was added. Functionality of endothelial cells differentiated from human iPSCs was demonstrated by primary vascular plexus formation upon co-culture with iPSC-derived pericytes and by incorporation in the vasculature of zebrafish xenografts in vivo [52, 66].

As vascular progenitors and in particular endothelial lineage cells in mammalian development emerge from the lateral and posterior mesoderm, and canonical WNT signaling was shown to play a decisive role in mesoderm commitment during embryogenesis, Patsch et al. investigated selective GSK3β inhibitors that promoted efficient commitment of human iPSCs towards mesoderm prior differentiation into endothelial cells [50]. WNT signaling directs differentiation of iPSCs into mesoderm and GSK3β inhibition activates this pathway. Thus, GSK3β inhibition and BMP4 treatment rapidly committed iPSCs to a mesodermal fate and subsequent exposure to VEGF resulted in the differentiation of mature endothelial cells with efficiencies over 80% within 6 days. Herein, CP21 and CHIR were the most selective GSK3β inhibitors. For differentiation, human iPSCs plated as single cells were treated with CP21 or CHIR and/or BMP4 for 2 days to foster mesodermal induction and then treated with VEGF to induce the endothelial cells. At day 5, endothelial cell differentiation was confirmed by analyzing the expression of VE-Cadherin via FACS and revealed that only BMP4 and CP21 or CHIR combined were capable of inducing high levels of endothelial cell marker expressing cells (up to 35% VE-Cadherin-positive cells), whereas treatment with BMP4 alone led to fewer than 10% VE-Cadherin-positive cells. A additional combined VEGF treatment with Forskolin (a potent cyclic AMP inducer known to activate kinase A resulting in an increase in vascular development) for 2 days, followed by VEGF treatment alone for additional 4 days promoted the differentiation of iPSCs into endothelial cells with efficiencies between up to 90% of cells. MACS-mediated selection for VE-Cadherin expressing endothelial cells then obtained virtually pure cultures [50].

The efficient and robust differentiation of endothelial cells from human iPSCs via lineage control with VEGF in combination with cyclic AMP was further stressed by a recent study of Ikuno et al. which demonstrated that cyclic AMP synergistically enhanced the VEGF effects in serum-free 2D monolayer cultures [67]. The authors controlled the direction of differentiation from mesoderm to endothelial cells using stage-specific stimulation with VEGF and cyclic AMP combined with the elimination of non-responder cells at early endothelial stages. This stimulation-elimination method robustly achieved very high efficiency (up to 99%) with no need of purification of endothelial cells after differentiation [67].

Very recently, Harding et al. developed a robust protocol to differentiate human iPSCs into endothelial cells with high purities in 8 days without cell sorting [68]. Here the protocol started also with the induction of mesoderm formation from iPSCs using the GSK3 inhibitor CHIR99021 for 2 days, which activated the WNT signaling pathway. A further treatment of iPSC-derived mesoderm with VEGF, BMP4, or FGF2 individually or in combinations for 2 days for the induction of vascular progenitors differentiation revealed that VEGF alone only moderately induced the expression of endothelial lineage markers such as VE-Cadherin and CD31. The combination of BMP4 and VEGF further increased the number of cells expressing CD31 and VE-Cadherin, whereas FGF2 alone failed to do so. However, the highest number of cells being positive for endothelial lineage markers was achieved with the combination of VEGF, BMP4, and FGF2, which was significantly higher than those with only VEGF and BMP4 combined [68]. The vascular progenitors were further grown in endothelial-specific growth medium (ECGM-MV2) supplemented with additional VEGF for 4–6 days yielding ~ 95% CD31-positive and ~ 80% VE-Cadherin-positive endothelial cells. Functionality of those iPSC-derived endothelial cells was demonstrated by their ability to form patent blood vessels that were connected to the host vasculature in the ischemic limbs of immune-deficient mice.

Another straight forward—but up to now quite theoretical—strategy would be the ectopic overexpression of endothelial related transcription factors (TF) in human iPSCs to generate endothelial cells. Especially FOX and ETS transcription factors are potent regulators for vascular development and angiogenesis, that regulate almost all typical endothelial markers [69–71]. Although a couple of endothelial-specific TF were identified, introduction of these factors to generate endothelial cells were restricted to somatic cell types, in particular fibroblasts of different origins [69, 72, 73]. Major concerns about using the target cell-specific TF for direct cell lineage reprogramming are: (1) to establish stably proliferative endothelial populations, a more precise temporal control on gene overexpression is needed and (2) the original gene regulatory network of the starting cell type may be insufficiently inactivated in the differentiated population [74]. Thus, it remains elusive whether the forced expression of selected endothelial cell-specific TF in human iPSCs would result in endothelial cell differentiation as already demonstrated for murine mouse embryonic stem cells [75, 76]. From murine iPSCs it is further known that specific micro-RNA (miR) regulate endothelial lineage emergence from differentiated iPSCs [44]. For example, miR-21 was one of the most overrepresented miR expressed, when murine embryonic stem cells were differentiated into endothelial cells and miR-199 was increased in a step-wise fashion when murine embryonic stem cells differentiate into endothelial cells [49, 77]. However, corresponding investigations using human iPSCs remain again elusive.

## Endothelial cell heterogeneity

In general, as shown by the numerous studies listed above, the isolation of endothelial cells primarily relies on general endothelial surface markers such as KDR/VEGFR2, CD31/PECAM1, or CD144/VE-Cadherin, which in turn means that generated endothelial cells might represent different endothelial subtypes: arterial, venous, and lymphatic endothelial cells. Endothelial cells exhibit a high degree of heterogeneity across vascular classes and tissue types, e.g. micro-/macro-vascular endothelial cells and sinusoidal endothelial cells [48, 78, 79]. Therefore, Rufaihah et al., investigated if endothelial subtypes can be enriched upon iPSC-endothelial cell differentiation [78]. The authors used HUF4 and HUF5 iPSCs generated from adult dermal fibroblasts to demonstrate that the specification of arterial or venous subtype upon endothelial cell differentiation from iPSCs is influenced by the concentration in the media of VEGF. Herein, high VEGF-A concentrations (~ 50 ng/ml) and 8Br-cAMP (8-bromoadenosine-3′:5′-cyclic monophosphate sodium salt) induced arterial CD31-positive endothelial cells in association with increased expression of the Notch pathway, whereas lower VEGF concentrations (~ 10 ng/ml) induced venous CD31-positive endothelial cells. In contrast, high VEGF-A and VEGF-C concentration with the supplementation of angiopoietin 1 in the medium promoted the specification to lymphatic CD31-positive endothelial cells [78]. Interestingly, iPSC-derived endothelial cells of the arterial subtype showed the best potential to form extensive and mature capillary networks in vivo, clearly indicating that refining the differentiation methods can enrich for subtype-specific endothelial cells upon iPSC differentiation with functional benefits of enhancing neovascularization. Although, focusing on human embryonic stem cells only, Sriram et al. also reported the efficient differentiation of human embryonic stem cells to arterial and venous endothelial cells under feeder- and serum-free conditions using different VEGF concentrations, which offers a human platform to study arterial-venous specification for the various applications in the future [79].

Conclusively, robust protocols exist to differentiate human iPSCs successfully to endothelial cells that have great potential for mechanistic studies of endothelial cell differentiation as well as for individualized vascular therapy.

## Reprogramming to SMC

Vascular SMC are crucial for vascular function providing contractile function and structural support to vascular blood vessels. SMC with more than one developmental origin still retain considerable plasticity and can modify their phenotype in response to vessel injury [80–82]. Most vascular SMC are largely derived from various mesodermal lineages such as splanchnic mesoderm, lateral plate mesoderm, and somatic or paraxial mesoderm and a subset of SMC originate from the neural crest, the secondary heart field and the pro-epicardial organ [81, 83–85]. Thus, many human iPSC-SMC differentiation protocols direct the cells toward an intermediate, origin-specific lineage before inducing the terminal SMC phenotype [86, 87].

Today, various embryonic stem cell-based differentiation strategies exist to induce in vitro differentiation of human iPSCs to SMC. In line with the protocols of differentiating human iPSCs to endothelial cells two major methods exist: (1) EB-induced differentiation with or without additional growth factor treatment to foster mesodermal induction, and (2) monolayer culture of iPSCs on extracellular matrix (ECM) coatings and by the use of defined chemical conditions followed by culture manipulations [31]. SMC differentiated from iPSCs might be further sorted using flow cytometry or immunomagnetic separation or enriched using selection medium to get pure SMC. With respect to the multiple origins of SMC during development, differentiating via the EB method might have an advantage of mimicking early embryonic development.

One of the first described EB-methods for differentiating SMC from human iPSCs was reported in 2010 when Lee et al. generated iPSCs from somatic human aortic vascular smooth muscle cells and differentiated these iPSCs then back into SMC [88]. Here, generated iPSC were cultured on mitomycin C-treated mouse embryonic fibroblasts as feeder cells in standard human embryonic stem cell medium. For SMC differentiation EBs were transferred to gelatin-coated plates and maintained in SMC-specific medium (SMCM) for 10 days. Colonies with SMC-like cell morphology were then manually picked, examined for the expression of SMC-specific marker genes, and further expanded [88].

In a modified EB-initiated differentiation method based on a protocol established for human embryonic stem cells, Ge et al. differentiated SMC from human iPSC clones which were established from foreskin fibroblasts and vascular SMC of patients with supravalvular aortic stenosis [89, 90]. Herein 6 days EB were plated on gelatin-coated culture dishes and cultured with fresh differentiation medium (DMEM supplemented with FBS, nonessential amino acids, β-mercaptoethanol and l-glutamine). On day 6 the small clusters obtained were dissociated and transferred to Matrigel-coated plates in SmGM-2 media. After 1 week of culture, cells were again transferred to gelatin-coated culture dishes and cultured with the low FBS (5%) differentiation medium for at least 5 days to complete the differentiation. This quite simple method yielded SMC of mixed origin (mesoderm, ectoderm an/or endoderm-derived) in a purity of more than 95% as analyzed by calponin expressions [89].

A further more mesodermal-directed approach was reported by Lin et al. where L1 iPS and L2 iPS6 cells (human iPSC lines) reprogrammed from dermal fibroblasts cultured on mouse embryonic fibroblasts were used [91]. SMC were differentiated from 6 days EB which were pre-induced with VEGF and the WNT signaling inhibitor Dickkopf homologue 1 (DKK1) for 2 days. Activated leukocyte cell adhesion molecule ALCAM/CD166-negative (to exclude cardiomyocytes) were isolated via flow cytometry and further cultured with SMC-specific SmGM2 medium yielding over 90% smooth muscle actin (SMA/ACTA2)-positive SMC. A similar approach using 6 days EB pre-induced with VEGF and FGF2 for 2 days was reported within the same study. A successive prolonged culture as a monolayer for additional 14 days with VEGF and FGF2 yielded about 70% SMC. Finally, over 95% pure SMC were obtained after a negatively isolation for CD31-reactivity via flow cytometry [91].

## SMC heterogeneity

Somatic SMC display a wide range of morphological and functional characteristics that are best described as a spectrum bounded by predominantly synthetic and contractile phenotypes [92–94]. Accordingly, it was already shown that human iPSC-derived SMC could be guided to acquire either a synthetic or a contractile phenotype [94]. By monitoring the expression of SM-MHC and elastin, the authors demonstrated the possibility of generating synthetic or contractile phenotypes from different human iPSC lines with appropriate concentrations of SMC-inductive factors (namely PDGF-BB and TGFβ1) known to control these developmental steps in the early embryo and in adulthood. The human iPSC cell lines MR31 (derived from IMR90 fetal lung fibroblasts) and BC were grown on inactivated mouse embryonic fibroblast feeder layers in KO-DMEM supplemented with KOSR, GlutaMAX, FGF2, NEAA and β-mercaptoethanol. Differentiation into SMC was induced by plating the cells on collagen-IV-coated plastic in a differentiation medium composed of alpha-MEM, FBS, and β-mercaptoethanol for 6 days. Differentiating iPSCs were re-plated onto collagen-IV-coated plates in differentiation medium supplemented with PDGF-BB and TGFβ1 for additional 6 days (a total of 12 days) [94]. The long-term differentiation of these iPSCs in high serum (10% FBS) with PDGF-BB and TGFβ1 successfully induced the synthetic SMC phenotype with increased ECM protein expression and reduced expression of contractile proteins, whereas serum starvation (0.5% FBS) and PDGF-BB deprivation caused maturation towards the contractile SMC phenotype.

Recently, Yang et al. reported two novel iPSC-SMC differentiation protocols that yield SMC with predominantly contractile or synthetic phenotypes using human iPSCs that had been reprogrammed from human cardiac and dermal fibroblasts [86]. Both protocols begin by initiation of differentiation of human iPSCs (cultured in mTeSR medium on Matrigel-coated plates) using the GSK inhibitor CHIR99021 and BMP4 for 2 days. Differentiation into synthetic or contractile SMC began on day 3. Synthetic SMC were produced by culturing the cells with VEGF-A and FGF2 in RPMI1640 medium and 2% B27 (without insulin) from day 3 to 7, then with VEGF-A and FGF2 in RPMI1640 and 2% B27 (with insulin) from day 7 to 10, and with PDGF-BB and TGFβ1 in RPMI1640 and 2% B27 from day 10 to 14. Contractile SMC were produced by culturing the cells with VEGF-A and FGF2 in RPMI1640 and 2% B27 from day 3 to 7, and with PDGF-BB and TGFβ1 in RPMI1640 and 2% B27 from day 7 to 14. Flow cytometry analyses of SMA expression indicated that ~ 45% of the cells obtained with each protocol assumed an SMC phenotype. Additional purification was performed by maintaining the differentiated cells in 4 mM lactate RPMI1640 metabolic medium for 4–6 days [86]. Thus, each protocol could be completed in 2–3 weeks (including the 4–6 days metabolic selection period) and yielded SMC populations that are ~ 95% pure and remain phenotypically stable for at least 20 generations. In contrast to the contractile SMC, synthetic iPSC-derived SMC expressed less calponin, more collagen-I, and more connexin 43, were quite resistant to carbachol treatment while having higher cell migration and proliferation rates [86].

Of note, SMC from distinct anatomic locations were derived from different embryonic origins. This diversity was not specified in SMC generated via the EB method although a mesodermal developmental origin seemed to be obvious. However, these pioneering studies further proved the use of human iPSCs as an autologous cell source for patient-specific cell therapy [31]. Beside their potential for regenerative therapy, iPSCs have become an important tool for modeling and investigating human diseases, as well as for screening drugs [6]. Therefore, more defined protocols for generating human SMC subtypes of the distinct embryonic origins were needed for studying the influence of SMC lineage on the spatial specificity of vascular disease because the regional distribution of vascular diseases may partially be related to the inherent heterogeneity in SMC lineages.

In 2014, Cheung et al. showed in an very defined protocol how human pluripotent (embryonic) stem cells can be differentiated into distinct populations of SMC subtypes under chemically defined conditions [95]. With respect to the limitation that the present review focusses on iPSC-derived vascular cells the important protocol of Cheung et al. is included due to the fact that the protocol was also proven to work for human iPSCs. Jiao et al. used the Cheung protocol to successfully differentiate human iPSCs reprogrammed from human peripheral blood cells (PBMCs) to paraxial mesoderm- and subsequently to paraxial-derived SMC [96–98]. Furthermore, the successful generation of human iPSC neural crest-derived SMC was reported using the Cheung protocol [99]. Herein, the initial differentiation stage (day 0–5 or day 0–7) begins with the induction of three intermediate lineages: neuroectoderm (NE), lateral plate mesoderm (LM) and paraxial mesoderm (PM) [95]. Early mesoderm differentiation was started with a combination of chemically defined medium (CDM), containing polyvinyl alcohol (PVA), FGF2, the strong inhibitor of phosphoinositide 3-kinases (PI3K) LY294002, and BMP4 for 1.5 days. Consequently, either LM differentiation was started in CDM-PVA medium with FGF2 and BMP4 for 3.5 days or PM differentiation in CDM-PVA medium with FGF2 and LY294002 for 3.5 days. To induce NE differentiation, cells were cultured in CDM-PVA medium with FGF and the potent inhibitor of the TGF-β/Activin/Nodal pathway that inhibits ALK5 (SB431542) for 7 days. Successfully intermediate lineage differentiation was confirmed by the presence of the characteristic markers (PAX6, Nestin, GBX2 for NE; KDR, NKX2-5, ISL1 for LM; TCF15, TBX6, MEOX for PM) and was followed by SMC differentiation: LM-, PM- and NE-cells were re-suspended as single cells in CDM-PVA medium containing PDGF-BB and TGFβ1 for 6 days. The cellular markers generally expressed by mature SMC Transgelin (Tagln/SM22α), calponin (CNN1), and smooth muscle myosin heavy chain (MYH11) as well as the exhibited well-defined fibrous morphology especially in mature SMC confirmed the generation of functionally distinct SMC [95, 100].

The differentiation of human iPSCs towards SMC through the mesoderm in general was investigated by Patsch et al. who showed that selective GSK3 inhibitors combined with BMP4 efficiently activated WNT signaling which in turn directed differentiation of iPSCs into mesoderm as described above for iPSC-derived endothelial cells [50]. The authors demonstrated that replacing endothelial-inductive cues with factors that promote SMC formation (Activin A and PDGF-BB) efficiently generated SMC. Thus, Activin A and/or PDGF-BB were added following mesoderm induction. This modification resulted in the formation of almost exclusively CD140b/PDGFRB-positive SMC with virtually no VE-Cadherin-positive endothelial cells detectable, when Activin A and PDGF-BB were used. These iPSC-derived SMC cultures were nearly homogeneous they required no further purification [50].

Collectively, these data demonstrate that SMC can be efficiently differentiated from human iPSCs, and that reliable protocols exist to generate SMC developmental origin-specific. Patient-specific SMC might facilitate the study of disease mechanisms and development of novel therapeutic interventions.

## Cell culture limitations

Although endothelial cells as well as SMC could be isolated simultaneously from small vessel biopsies and expanded in culture, the quality of the cells after expansion is still not clear and primary isolates were prominently characterized by a very limited proliferation potential [101–103]. Although a genetic manipulation of endothelial cells and SMC by the introduction of human telomerase reverse transcriptase subunit resulted in prolonged proliferation potential while characteristics of normal control cells were retained, safety of the cells after genetic manipulation is still a great concern [104, 105]. Human iPSCs-derived vascular wall cells might be the promising way to solve the cell proliferation problem.

However, Li et al. performed a functional characterization of human iPSCs (derived from IMR90 fetal fibroblasts) and embryonic stem cell (H9)-derived endothelial cells and reported that iPSC-derived endothelial cells proliferated at a lower rate than endothelial cells differentiated from embryonic stem cell [106]. Herein, iPSC-derived endothelial cells were characterized by a lower G2/M phase population and a downregulation endothelial marker proteins upon prolonged culture, indicating that CD31-expressing endothelial cells from human iPSCs cannot be readily expanded in vitro, although the vasculogenic potential was not affected. A global gene and miRNA profiling of human iPSC- and embryonic stem cell-derived endothelial cells demonstrated a high degree of similarity between the generated endothelial cells, in particular as compared to human umbilical vein endothelial cells, although miRNA profiles were significantly different in respective iPSCs and embryonic stem cells [107]. However, epigenetic instabilities and/or phenotypic switches (e.g. spontaneous endothelial-to-mesenchymal transition) after prolonged culture might occur because the endothelial identity may not be well inherited in human iPSCs- and embryonic stem cells-derived endothelial cells [58, 106, 108]. Human iPSC-derived hemangioblasts, endothelial cells, and hematopoietic cells were already shown to bear similar phenotypic and morphologic characteristics to those derived from embryonic stem cells, but generation of respective cells was less efficient [109]. Prolonged culturing further demonstrated severely limited growth and expansion capabilities, early cellular senescence and significantly increased apoptosis in iPSC-derived vascular cells. The latter ones could not be observed in iPSC-derived endothelial cells investigated by Li et al., which might be due to the different differentiation methods that were used [106, 109]. Moreover, the observed differences might be based in the different cellular origins used for iPSC generation, because some reports already revealed that a somatic memory does exist [110–112]. In line with this hypothesis, it was also shown that the cellular origin influences the lineage differentiation propensity of human iPSCs [113]. Endothelial cells differentiated from iPSCs derived from three types of somatic cells (fibroblasts, endothelial cells and cardiac progenitor cells) of the same individuals showed clear differences in the endothelial cell differentiation propensity and gene expression of endothelial-specific markers. In vitro and in vivo endothelial cells differentiated from endothelial cell-derived iPSC maintained a higher CD31-positive population upon long-term culturing and showed higher recovery rates than those derived from fibroblasts and cardiac progenitor cells [113].

Thus, the somatic memory of iPSCs should be carefully considered before clinical translation. Furthermore, improvements in the long-term culture are needed to facilitate the clinical application of human iPSC-derived vascular cells and in particular endothelial cells in the future.

## Applications

iPSCs are capable of self-renewal and differentiation into any cell type in the human body, and thus are attractive resources to generate unlimited numbers of vascular cells. Many groups have shown that vascular cells and in particular endothelial cells and SMC could be reproducibly derived from human iPSCs. Human iPSC-derived vascular cells have become of interest as patient-specific therapeutic tools. The respective mechanistic studies may gain knowledge that aim in understanding the pathogenesis of vascular disease, foster developing interventions and therapeutics as well as enabling drug screening applications. The in vitro vascular cell differentiation from iPSCs also provides a new opportunity to study molecular mechanisms that control endothelial cell or SMC fate. These cells represent a potentially valuable tool and for regenerative therapies, e.g. cell replacement to re-vascularize ischemic areas and the development of robust and reproducible vascular tissues (stem cell-based vascular engineering).

## Patient-derived vascular cells: unraveling mechanisms and disease modeling

Abnormal proliferation of vascular SMC can lead to narrowing or blockage of the ascending aorta and other arterial vessels. This disease pattern is characteristic for supravalvular aortic stenosis (SVAS) which is caused by mutations in the elastin gene [89]. Using a modified EB-initiated differentiation method (for protocol see above) to generate SMC from human iPSCs reprogrammed from foreskin fibroblasts and from epicardial coronary arteries SMC of patients with SVAS, the authors validated that SMC derived from SVAS iPSCs have the prototypical hyper-proliferation response seen in primary SMC from SVAS patients. Furthermore, novel evidence was provided that ERK1/2 signaling is activated in SVAS iPSC-SMC and accounted for their hyper-proliferation, which suggested that inhibition of cellular proliferation by decreasing ERK1/2 signaling might be potential therapeutic strategies in SVAS patients [89]. Conclusively, SVAS iPSC-SMC recapitulated key pathological features of patients with SVAS and may provide a promising strategy to study disease mechanisms and to develop novel therapies. In a further study using SMC differentiated from SVAS-iPSCs the authors nicely showed that these that in line with the increased migration potential, these SVAS iPSC-SMC have robustly up-regulated expression of integrin-β3 protein, strongly indicating that enhanced integrin-β3 signaling builds a crucial link between elastin deficiency and arterial hyper-muscularization. Thus, β3-blockade is a promising and much needed noninvasive therapeutic approach for SVAS [114].

As it is already known that vascular SMC subtypes show lineage-specific differences in growth, gene expression, and functional properties, and that these differences may contribute to site-specific patterns of vascular diseases (such as aortic aneurysm), the progress in defining protocols for generating human SMC subtypes of distinct embryonic origins already contributed to dissect cell-type specific molecular mechanisms. Human iPSC-derived embryonic lineage-specific SMC were used to gain insight into the molecular mechanisms involved in SMC development. NOTCH3 signaling emerges as one of the key regulators of vascular SMC differentiation and maturation in vitro and in vivo in a lineage- and temporal-dependent manner [81]. The authors followed the Cheung protocol and used SMC differentiated from the three intermediate populations (neural crest, lateral and paraxial mesoderm) of human iPSCs and profiled the expression of NOTCH receptors, ligands, and downstream elements during the development of origin-specific SMC subtypes. NOTCH3 was the only NOTCH receptor to show lineage-specific expression changes during SMC development. In addition, NOTCH3 and its ligand, JAG1, share a similar expression trend in developing and mature SMC [81].

Bargehr et al. investigated the respective potential of different embryonic origin-specific SMC derived from human embryonic stem cells to support endothelial network formation in vitro using their previously generated Cheung protocol of lineage-specific SMC [115]. Lateral mesoderm-derived SMC were shown to support endothelial network complexity and survival in three-dimensional co-culture via increased expression of Midkine/neurite growth-promoting factor 2 as one important mediator for the enhanced vasculogenic potency, further highlighting a lineage-specific approach being beneficial for vascular tissue engineering and therapeutic revascularization [115].

Understanding the regulatory mechanisms that control SMC differentiation from vascular progenitors with respect to their developmental origin is essential for exploring therapeutic targets for potential clinical applications because differences in embryological origins between SMC could contribute to site-specific localization of vascular diseases [80, 82, 100, 116]. The recent advancement of iPSCs and lineage-specific SMC differentiation technology now provides unique methods to investigate human SMC subtypes of distinct embryonic origins on the spatial specificity of vascular disease. For example, the aortic root, ascending aorta and aortic arch were shown to be populated by SMC arising from neural crest, while the descending aorta is populated by SMC from the paraxial mesoderm. Jiao et al. used the Cheung protocol to investigate whether the aortopathy in patients with congenital cardiovascular malformation (bicuspid aortic valves, BAV) is due to a defective differentiation of neural crest stem cells to SMC that spares the paraxial mesoderm cells-derived SMC. It is known that aneurysms associated with BAV most commonly involve the aortic root, aortic arch, and ascending aorta while spare the descending aorta [96]. Interestingly, neural crest-derived SMC generated from iPSCs (reprogrammed from PBMC) from patients bearing BAV/thoracic aortic aneurysms and not paraxial mesoderm-derived SMC were shown to contribute to the aortopathy associated with BAV because these cells showed a decreased differentiation and contraction potential [96].

The growth and reactivity of SMC were influenced and controlled by the underlying endothelial cells that comprise a permeable tissue layer, which controls the interaction of the vessel wall with circulating blood components, and regulates the vascular response to hemodynamic forces. Collado et al. speculated that iPSC-derived endothelial cells and SMC represent an immature phenotype because these cells were not derived from an intact blood vessel [99]. Because hemodynamics and heterotypic cell–cell communication play important roles in vascular cell phenotypic modulation the authors co-cultured iPSC-derived endothelial cells and SMC under vascular region-specific blood flow hemodynamics, and compared these hemodynamic co-cultures with adult blood vessel-derived endothelial and SMC. Their studies revealed that the hemodynamic co-culture restored a high degree of similarity in their responses to pathological stimuli which were associated with vascular diseases (e.g. atheroprone hemodynamics which promote inflammation, monocyte transmigration, differentiation and ensuing atherosclerosis) [99]. Thus, iPSC-derived vascular cells exposed to hemodynamics may provide a more-realistic viable system for modeling rare vascular diseases and testing new therapeutic approaches. Belair et al. used iPSC-derived endothelial cells in combination with the well known angiogenic tube formation assay on Matrigel (3D vascular network formation) to quantitatively assess capillary-like structures in response to growth factor stimulation and pharmacological inhibition [117]. This combination demonstrated the feasibility of using a well-defined, stable source of iPSC-derived endothelial cells to model blood vessel formation within a variety of contexts formats that enables basic biological research and molecular pharmacology studies with the potential to generate donor specific disease models.

Human iPSC-derived endothelial cells cultured in peptide-functionalized poly(ethylene glycol) (PEG) hydrogels, either on standard well plates or within a passive pumping polydimethylsiloxane tri-channel microfluidic device were further shown to offer a defined platform for investigating vascular morphogenesis in vitro using both standard and microfluidic formats [118]. These PEG hydrogels contained incorporated matrix metalloproteinase (MMP)-degradable crosslinks and the synthetic peptide Gly-Arg-Gly-Asp-Ser (GRGDS) (which corresponds to a fragment of fibronectin and contains its cell adhesion sequence RGD) that caused degranulation and spreading of monolayers cells. The authors showed that iPSC-derived endothelial cells self-assembled into vascular networks through physiologically relevant mechanisms when cultured in these PEG hydrogels, and capillary tubules with lumens were stable for at least 2 weeks when the hydrogels were polymerized within the microfluidic device. These results again demonstrated the value of engineering approaches for extending the stability of in vitro vascular networks to form robust and reproducible vascular tissues using iPSC-derived endothelial cell monocultures for disease modeling and screening applications [118].

Concerning the stability of developing 3D microvascular networks, which were build up of endothelial cells, Kurokawa et al. confirmed that human iPSCs-derived endothelial cells formed stable, perusable micro-vessels when cultured within 3D microfluidic devices [119]. Again, it was shown that iPSCs-derived endothelial cells comprise similar physiological functions characteristic of primary endothelial cells in a series of in vitro assays including permeability, response to shear stress, and the expression of endothelial markers with reproducibility. Thus, micro-physiological systems (or organ-on-a-chip) platforms in combination with iPSCs-derived endothelial cells were well suited to recapitulate in vivo physiology using small-scale in vitro tissue models of human physiology.

Human iPSC-derived endothelial cells from three families with familial pulmonary arterial hypertension (FPAH) patients, where the autosomal dominant disease-causing BMPR2 mutation is only 20% penetrant, unaffected mutation carriers, and gender-matched controls were compared to investigate modifiers of BMPR2 signaling [120]. FPAH patient derived iPSC-endothelial cells showed reduced adhesion, survival, migration, and angiogenesis compared to the cells derived from unaffected mutation carriers or from controls. Protective BMPR2 modifiers were shown to preserve pP38 signaling and adhesion in iPSC-derived endothelial cells of unaffected mutation carriers, identified BIRC3 (an inhibitor of apoptosis) and correlated increased BIRC3 expression levels to normalization of EC survival. Finally, the correction of the BMPR2 mutation in FPAH patient derived iPSC-endothelial cells restored signaling and EC function, which suggested that identified protective modifiers for FPAH that could help to inform the development of future treatment strategies [120].

Tseng et al. differentiated endothelial cells from human iPSCs which were reprogrammed from peripheral blood cells of patients with Fabry disease to investigate specifically the accumulation of globotriaosylceramide (GB3) in endothelial cells, which is considered to be pathogenically responsible for the phenotype variability of the disease that causes cardiovascular dysfunction [121]. Fabry disease is an X-linked inherited lysosomal storage disease caused by α-galactosidase A (GLA) deficiency resulting in the accumulation of GB3 in a variety of tissues, but recent attention shifted towards studying the mechanisms through which Gb3 accumulation in vascular cells leads to endothelial dysfunction and eventually multi-organ failure, while the underlying mechanism remains elusive. Using endothelial cells derived from iPSCs Fabry diseased patients in comparison to healthy control iPSC-derived endothelial cells, the authors identified superoxide dismutase 2, a mitochondrial antioxidant, to be significantly downregulated in the patient-derived endothelial cells. Together with an increased production of reactive oxygen species (ROS) and a significantly enhanced AMPK activity, finally vascular endothelial dysfunction was caused, suggesting dysregulated mitochondrial ROS may be a potential target for treating Fabry disease in particularly for severe Fabry disease-associated cardiovascular complications [121]. Using endothelial cells differentiated from mesoderm-induced human iPSCs Harding et al. revealed that all three MAPK (ERK, P38, and JNK) and the PI3K pathways are responsible for the induction of endothelial cell fate [68]. Furthermore, their generated homogeneous endothelial cells could be engrafted and generated functional vasculature in normal and ischemic environments in vivo. Conclusively the authors provided evidence that iPSC-derived endothelial cell have great potential to treat a vascular disease in particular with respect to the development of cell therapy to treat ischemia.

Reprogrammed skin fibroblasts from two Hutchinson–Gilford Progeria syndrome (HGPS) patients and two healthy HGPS parents were differentiated to vascular cells to investigate the impact of the substance progerin on the functional properties of the different cell types [122]. HGPS is caused by progerin (a truncated and farnesylated form of Lamin A) and affects mesenchymal lineages, including the skeletal system, dermis, and vascular SMC. Using HGPS-iPSC-derived SMC the authors identified cytosolic heterogeneously sized, calponin-positive inclusion bodies in the cytoplasm that affected the contractile properties of the SMC in situ. As autopsies already indicated that HGPS-related death is associated with premature atherosclerosis which may be accompanied by vascular SMC loss, the authors further reported a pronounced sensitivity of the HGPS-SMC to various imposed insults (hypoxia, combination of hypoxia and substratum deprivation, repeated pulses of electrical stimulation). This work was not only one of the first reports of an iPSC-based disease model of HGPS, together with the revealed perturbation of contractile properties due to calponin sequestration, this lineage may feature prominently in the pathology of progeria [122].

Another model to dissect the molecular mechanisms of a certain disease and thereby allowing to identify novel targets for treatment and to provide an innovative human platform for the testing of new drugs is the iPSC-derived vascular model of Marfan syndrome that was used to identify key mediators of SMC death [97]. The authors followed the Cheung protocol to generate embryonic lineage-specific SMC which enabled them to recapitulate the pathology seen in Marfan aortas, including defects in fibrillin-1 (FBN1) accumulation, ECM degradation, TGFβ signaling, contraction and apoptosis finally resulting in aortic aneurysms in Marfan syndrome patients. Within these model Granata et al. highlighted the role for p38 MAP kinase in regulating SMC apoptosis and proliferation and identified KLF4 as a potential contributing factor to Marfan syndrome pathology. The detected abnormalities, like upregulation of both P-p38 and KLF4 observed in the iPSC-derived SMC as well as the detected high TGFβ levels were further corrected by CRISPR-based editing of the FBN1 mutation [97]. The authors even validated their findings in patient samples, and thus confirmed that the in vitro system accurately modeled the human Marfan disease. Thus, this work further proved the use of iPSCs as model cells representing a tool to dissect molecular and mechanosensing mechanisms of certain diseases and thereby including the ability to identify novel targets for potential treatments, as well as a resourceful platform for testing new drugs.

## Drug screening

iPSC-derived modeling systems offer a dual capacity for investigators because they cannot only offer a platform for gaining valuable insights into the mechanisms of disease progression but can also be used as a tool for drug screening to treat the disease [6, 99].

Patient-derived iPSCs were already used as potential source for studying anti-hypertensive drug response [123]. Therefore, multiple iPSC lines generated from PBMCs isolated from hypertension patients were established and vascular SMC were differentiated via EB-generated mesenchymal intermediates in the presence of SMC-specific factors for 10 days. The contractility of the generated and FACS-sorted (CD140b/PDGFRB- and CD91/LRP1-positive) SMC and the inflammatory signaling was investigated in response to different chemical (Phorbol 12-myristate 13-acetate, PMA) or physiological stimuli (endothelin-1) was analyzed, and nicely showed that all iPSC-SMC lines contracted and tremendously responded to different inflammatory stimuli [123]. With respect to hypertension, pharmacogenomics studies seek to identify genetic sources of variable anti-hypertensive drug response. Up to now this study did not unravel potential relationships between drug responses and genetic associations, namely single-nucleotide polymorphisms, but these investigations using vascular cells generated with iPSC technology paved the way for providing a great interface to bring patient cells with their genomic data into the laboratory and to study hypertensive responses [123].

A platform based on endothelial cells derived from iPSCs generated from human cord blood was established for drug screening from Vazao et al., which may open new avenues of research for the study and modulation specifically of the embryonic vasculature [124]. Herein, generated iPSCs were differentiated into embryonic endothelial cells, allowed to mature under flow conditions for more accurate toxicological assessment prior exposing the cells in static conditions to a Library of Pharmacologically Active Compounds (LOPAC, from Sigma). The library consisted of 1280 pharmacologically active compounds, which were inhibitors, receptor ligands, pharma-developed tools, and approved drugs that impacts most signaling pathways and covers all major drug target classes. Interestingly, two compounds that had higher inhibitory effect in embryonic than postnatal endothelial cells were identified: fluphenazine (an antipsychotic), which inhibits calmodulin kinase II and pyrrolopyrimidine (an anti-inflammatory agent), which inhibits VEGFR2, decreases endothelial cell viability, induces an inflammatory response, and disrupts preformed vascular networks. The vascular effect of the pyrrolopyrimidine was further validated in embryonic and adult zebrafish [124]. As the vascular system is the first functional organ to develop in the mammalian embryo, and the disruption of the vascular system has been correlated with fetal loss, human malformations, and cognitive impairment, these generated platform is important for the identification of compounds with developmental toxicity. Conclusively the authors achieved a huge effort in generating a valid alternative to animal testing that be used to screen existing and newly developed drugs.

Up to now, patient-specific iPSCs have become an important tool for investigating and modeling human diseases, as well as for screening drugs.

## Treatments/vascular grafts

The blood vessel system is a dense vascular network that forms the transport routes for nutrients, oxygen, hormones and much more simultaneously disposes of toxic waste from the body. However, as soon as something in the vessels is not correct, so that we suffer from vascular disease, the affected person quickly notices signs of illness or failure. Thus, the blood vessels play a decisive role when it comes to the functional and viability of all organs and organs. Like all other organs, the blood vessels are not indefinitely resistant to all external and internal influences. Especially in the present time, diseases of the blood vessel system are increasing to an astonishing extent. Disorders of the blood vessels and the heart are grouped as cardiovascular diseases [125, 126]. Cardiovascular diseases remain the leading cause of death in the world, accounting for 17.7 million premature deaths (under the age of 70) per year worldwide in 2015, representing 31% of all global deaths [127]. Depending on the anatomical region of involved blood vessels, cardiovascular diseases include coronary heart disease (blood vessels supplying the heart muscle), cerebrovascular disease (blood vessels supplying the brain), peripheral arterial disease (blood vessels supplying the arms and legs) and deep vein thrombosis and pulmonary embolism (blood clots in the leg veins, which can dislodge and move to the lungs) [125, 126].

The current standard of care to treat patients which suffer from coronary heart disease in the need of conduit vessels and bypass grafts is the use of autologous donor vessels as vascular grafts [127–129]. However, availability of healthy autologous vessels is often limited from these patients, which is mainly based to existing endothelial dysfunction [128, 130, 131]. In addition, recruitment of monocytes causing neointimal hyperplasia can be induced resulting from damage to the delicate endothelium during vascular invention [132, 133]. Also mechanical integrity of these vascular scaffolds might be impaired, possibly due to a lack of mature SMC, and the addition of SMC has been shown to regulate endothelial cell function by increasing expression of angiogenic factors [134–136]. Although commercial polymeric bypass grafts based on synthetic materials such as polytetrafluoroethylene or polyurethane are a suitable alternative for patients who lack suitable donor vessels, they do not support long-term patency as a small-diameter vascular graft [130, 137–139]. Thus, the ultimate goal of vascular tissue engineering is to develop cell-based small-diameter vascular grafts that resist thrombosis and support graft patency by generating biologically based vascular grafts that exhibit the biological and mechanical properties of native arteries [139, 140].

Samuel et al. generated functionally competent and durable engineered blood vessels from human iPSC-derived endothelial cells [141]. The authors investigated the vasculogenic capacity of endothelial cells derived from several reprogrammed fibroblasts (human adult dermal fibroblast cell line HFib2-iPS4, HFib2-iPS5, human foreskin fibroblast cell line HS27-iPSC, and human foreskin fibroblast cell line BJ-iPSC cells) which were purified by flow cytometry sorting using combinations of anti-CD34, KDR, and NRP1 antibodies. In combination, a model of durable blood vessel formation in vivo in mice using the murine embryonic precursor cell line 10T1/2 as supporting perivascular cells was used [142]. Generated endothelial cells from all iPSCs formed stable functional blood vessels in vivo, lasting for around 280 days in mice [141]. Furthermore, the authors successfully generated functional blood vessels in vivo using endothelial cells derived from three different iPSC lines reprogrammed from fibroblasts of type-1 diabetic patients. Beside the well-known metabolic impairments, diabetes is characterized by a prominent endothelial dysfunction (e.g. augmented vasoconstriction, increased inflammation and thrombosis) of many vascular beds, directly affecting wound healing processes in diabetic patients [143, 144]. Therefore, diabetes is considered as a vascular disease and a therapeutically improvement of endothelial dysfunction is a major issue in the prevention of vascular complications associated with all forms of diabetes mellitus [143, 145, 146]. Beside the potential of an autologous therapy approach using reprogramed iPSCs-derived vascular cells with reversed stress effects of high glucose caused by diabetes, the generation of vasculatures holds great promise in treating diabetic patients with vascular and wound healing complications. Supporting evidence was already investigated by Chan et al., who differentiated early vascular cells from type 1 diabetes mellitus (T1D) patient-derived hiPSCs, generated vascular networks of the respective matured endothelial cells in deliverable hydrogels and further demonstrated the integration into host vasculature networks in vivo [147]. Herein, vascular endothelial cadherin-positive cells (early endothelial cells) and platelet-derived growth factor β-positive cells (early pericytes) were differentiated from the T1D-derived iPSC cell lines T1D H2.1 and T1D 1018S, as well as from the healthy control donor cell line BC1 using high VEGF media treatment after mesodermal induction in iPSCs plated onto collagen IV coated plates. Upon maturation T1D-iPSC-derived early endothelial cells expressed mature endothelial markers and displayed behaviors typical of mature and functional endothelial cells, in contrast to T1D-derived endothelial (progenitor) cells that beside an overall reduction in the amount are characterized by an prominent dysfunctionality [148–150]. Functionality of T1D-iPSC-derived endothelial cells was further confirmed in response to hypoxia, which again highlights the potential of these cells as a therapeutic tool to treat diabetic vascular complications by the delivery of a microvascular bed e.g. in a hydrogel matrix [52, 147, 151]. Thus, this work points out nicely that patient-specific iPSC-derived vascular cells might provide a unique opportunity to advance autologous therapy for diabetic-vascular complications, in particular for diabetic wound healing.

As it is known that type-1 diabetic is associated with dysfunctional endothelial cells, capability to generate large amounts of functional endothelial cells from patients for autologous cell transplantation or tissue-engineering strategies is highly appealing for many diseases where re-vascularization is required [141, 152]. Hu et al. used SMC differentiated in the presence of TGFβ1 and PDGF-BB from of integration-free reprogrammed iPSCs out of human PBMCs for vascular tissue regeneration [153]. Generated SMC were successfully seeded on 3D macroporous nanofibrous poly(l-lactide) scaffolds, which had been coated with fibronectin prior subcutaneous implantation in immuno-deficient mice. Histological analysis of the implants after 14 days revealed that implants showed uniform cell growth inside the entire scaffold and significant collagenous matrix deposition, while no teratoma tissue formation was observed. Collectively this study had established an efficient patient-specific approach using iPSC-derived vascular cells towards in vivo regeneration of vascular tissue [153].

In 2015 already Ren et al. greatly reported the improved regeneration of functional pulmonary vasculature by repopulating the vascular compartment of decellularized rat and human lung scaffolds with human iPSC-derived endothelial cells and SMC [140]. Initially the authors successfully established transplantable rat lung grafts by delivering epithelial and endothelial cells, namely human umbilical cord venous endothelial cells into vascular and airway compartments of decellularized rat lung scaffolds [154]. However, the in vivo graft function declined within hours after transplantation, with substantial pulmonary secretions and the development of graft edema. These deficiencies were due in part to poor vascular performance of the engineered lungs. Endothelial coverage of scaffold vessels turned out being incomplete, which promotes blood clotting and hemorrhage [140, 154, 155]. When Ren et al. scaled their approach to the human lung lobe in combination with iPSC-derived endothelial cells, an efficient cell delivery, maintenance of cell viability and establishment of perfusable vascular lumens was achieved [140]. With respect to the aim of using autologous cells for clinical applications, the authors successfully regenerated lungs with vascular cells derived from human iPSCs that re-established of a functional vascular lumen across the entire graft with mature barrier function and sustained capillary perfusability.

Obtaining vascular SMC with robust, mature, elastic fibers is a key obstacle in tissue-engineered blood vessels. Eoh et al., followed the Wanjare protocol to differentiate SMC from human iPSCs and placed these cells on scaffolds in a pulsatile flow bioreactor, resulting in vascular smooth muscle tissue with robust elastic fibers and enhanced functionality [156]. Human iPSCs were dissociated and cultured on collagen type-IV-coated dishes in differentiation media (alpha-MEM with 10% FBS) for 6 days prior re-plating in differentiation media supplemented with TGFβ1 and PDGF-BB for additional 6 days. Differentiated smooth muscle-like cells were then seeded onto PEGdma-PLA [poly(ethylene glycol) dimethacrylate/poly(l-lactide)] scaffolds in quiescent media (containing TGFβ1 and 0.5% FCS) and either incubated under static conditions or subjected to peristaltic flow in a bioreactor system for the remainder of the differentiation (day 30) [156]. As SMC of the tunica media produce a complex ECM comprised of both elastin and collagen, which dictate the mechanical properties of blood vessels, significantly increased expression levels of ECM components (elastin, fibronectin, collagen I) were detected iPSC-smooth muscle tissue cultured in the bioreactor, which had further an increased calcium signaling and contraction in comparison to the static control. Thus, the authors presented an effective approach to engineer elastic functional vascular smooth muscle tissue for tissue engineering and regenerative medicine applications [156].

A straight forward approach concerning the clinical utility of iPSC-derived vascular cells was recently, reported by Lee et al. who enriched human iPSC-derived endothelial cells via a clinically compatible system [157]. Herein, a couple of human pluripotent stem cell lines (H1, H7, and H9 embryonic cells as well as BJ1 and PGP1 iPSCs) cultured in mTeSRTM 1 on Matrigel were differentiated towards the mesodermal lineage using CHIR99021. After 4 days endothelial cell differentiation was induced using medium containing VEGF-A, FGF2, EGF, DLL4, and heparin for additional 5–9 days. An additional treatment of the respective cultures with the Notch ligand, DLL4, further increased the cell numbers of cells expressing the endothelial marker KDR/VEGFR2/FLK1. Cells were purified then using MACS technology in combination with CDH5/CD144/VE-Cadherin expression and successfully implanted in an animal model of hindlimb ischemia. To finally enhance cell survival, vessel-formation, and the therapeutic potential generated cells were by encapsulated with a biocompatible peptide amphiphile (PA) nanomatrix gel prior to implantation. Corroboratively, the PA nanomatrix encapsulated iPSC-derived endothelial cells showed better perfusion recovery, more robust and longer cell survival, and higher and prolonged angiogenic and vascular incorporation capabilities for tissue ischemia after 10 month than the group which received the same cells without the matrix [157]. With respect to the critical importance for a clinical applicability, this work demonstrated nicely that (1) endothelial cells could be differentiated from human iPSCs under defined conditions free of xenogeneic components, (2) endothelial cells were yielded at high efficiency, (3) the procedure was verified in multiple cell lines, and (4) the therapeutic effect and safety (no teratoma formation) was verified in animal models of ischemic vascular disease.

## Vascularization strategies for other tissues

**Fig. 3:**
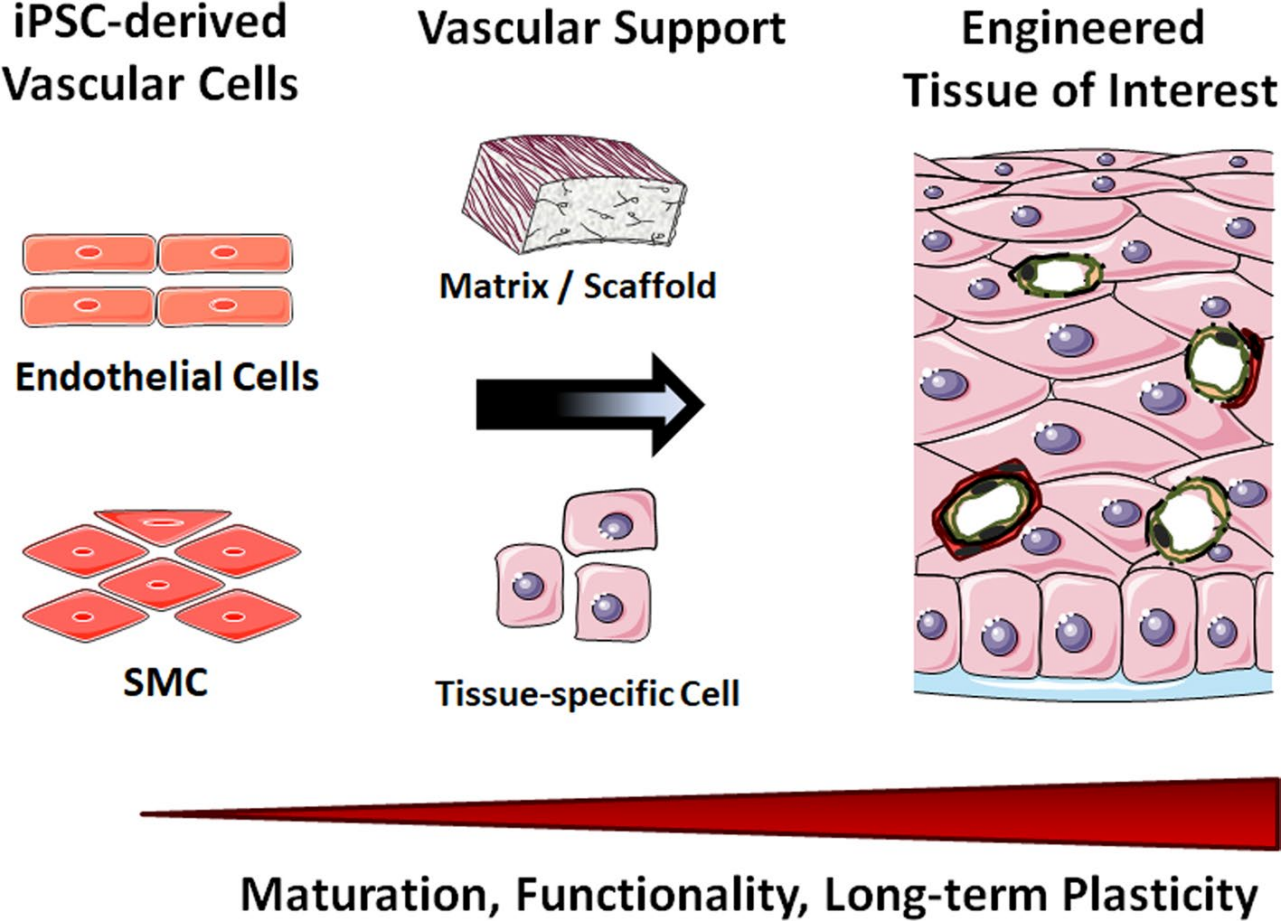
Producing tissue constructs with incorporated supportive vascular networks are required for functional regeneration of any tissue. Tissue engineering/regenerative medicine strategies utilize biocompatible matrices and scaffolds to provide structural support for the tissue-specific cells of interest that have the ability to form tissues in vitro or within the body upon transplantation. These tissue engineering matrices are designed to mimic the naïve situation, and thus to influence the physical, chemical and biological environment surrounding a certain cell population, e.g. hepatocytes, intestinal epithelial cells, skeletal or cardio-myocytes. Concerning oxygen diffusion and nutrient perfusion it is desired that a tissue is prevascularized before implantation, because generally the diffusion limitation allows only cells within 1–2 mm from the nearest capillary to survive. Co-culturing and integration of supporting vascular cells was further shown to improve maturation and functionality of the tissue-specific cells of interest, finally resulting in a better engraftment upon in vivo implantation

Clinical applications for vascular-related diseases (e.g. heart attack caused by atherosclerosis) commonly use the grafting of patients autologous arteries and veins, which are limited and often damaged due to the initial disease or advanced aging [158, 159]. Tissue-engineered replacement vessels represent an ideal solution to this clinical problem [158, 160]. Besides the direct forming of artificial blood vessels, producing tissue constructs with improved supporting vascular networks, as incorporation of a functional vascular network is required for functional regeneration of any tissue (Fig. 3). Thus, the creation of a stimulating microenvironment by the inclusion of vascular cells supports also various other tissue types, including engineered humanized intestinal grafts [161], livers [162, 163], and cardiovascular [164–166] and skeletal muscle tissues [167–170].

The supportive action of an endothelial cell co-culture with the generated cells of interest for organogenesis was already reported [165, 171, 172]. Takebe et al. generated hepatocyte-specific definitive endodermal cells from human iPSCs and cultured these cells than with human umbilical vein endothelial cells (HUVEC) resulting in an iPSC-derived organoid, the embryonic liver bud [163, 173]. After transplantation into immune deficient mice the iPSC-derived organ buds became directly connected to the host vasculature and showed the classical features of functional hepatocytes for a couple of weeks [163]. Human umbilical vein endothelial cells were also used to regenerate the endothelium necessary for a perfusable vasculature in engineered humanized intestinal grafts by repopulating de-cellularized rat intestinal matrix with human iPSC-derived intestinal epithelium [161]. Transplantation of these bioengineered intestinal grafts into immunodeficient rats further successfully showed (1) functionality by absorptive characteristics of the perfusable grafts and (2) survival of the bioengineered human intestinal tissue with preservation of basic function of the grafts following long-term heterotopic transplantation in vivo [161]. In line with these findings Valarmathi et al. used human cardiac microvascular endothelial cells together with human iPSC-derived embryonic cardiac myocytes in combination with a 3-D collagen cell carrier to generate an in vitro 3-D functional vascularized cardiac muscle construct [164].

Although non-immunogenic autologous endothelial cells as well as SMC isolated from patients themselves would be the first choice for vessel and/or vascularized tissue engineering, the numbers of cells obtained from small vessel biopsies remain limited and, moreover, these terminally differentiated cells bear a limited proliferation potential. Even the cells isolated from umbilical veins have limited proliferation potential [102, 174]. Thus, iPSC-derived vascular cells are an attractive source of cells that have been explored for the production of blood vessel replacements [175]. Conformingly, Giacomelli et al. generated and characterized human cardiac microtissues in vitro that integrated both cardiomyocytes and endothelial cells co-differentiated from human iPSCs in complex 3D structures after initial cardiac mesoderm induction [176]. This in vitro model was shown to recapitulate nicely the native human physiology because of its incorporated cardiomyocyte-endothelium crosstalk finally resulting in beating 3D structures 7 and 20 after initial aggregation and thus representing an advanced human stem cell-based platform for cardiovascular disease modeling and/or testing of relevant drugs [176]. Masumoto et al. successfully generated a novel 3D engineered cardiac tissue composed of human iPSC-derived cardiomyocytes, vascular endothelial cells, and mural cells that displayed excellent in vitro structural maturation and electromechanical performance translation [177]. Furthermore, implantation of these engineered cardiac tissues in an immune tolerant rat myocardial infarction model resulted in a proper regenerated myocardium at 4 weeks after implantation, demonstrating that the iPSC-derived engineered tissue comprised of cardiomyocytes and vascular cells were capable of in vivo survival, remodeling, and perfusion post-implantation with a chimeric composition of both host and graft-derived vasculature [177].

The integration of supporting vascular cells was further shown to improve maturation and functionality of engineered skeletal muscle tissue, finally resulting in a better engraftment upon in vivo implantation [167–170]. Criswell et al. showed that the addition of endothelial cells enhanced vascularization, innervation and muscle tissue formation when implanted muscle progenitor cells formed mature striated muscle fibers [168]. This effect was most likely related to VEGF because endothelial cell-derived VEGF secretion resulted in muscle progenitor cells migration and protection from apoptosis [168, 178, 179].

## Concluding remarks

The therapeutic potential of human iPSCs is quite promising as they are patient-specific stem cells that could be derived from easily accessible sources of tissue—such as the donor’s skin or peripheral blood—and that do not face the immunological barrier or ethical concern which confront cells derived from human embryonic stem cells. With the appropriate differentiation protocols, iPSCs could then be used to generate vascular cells. Thus, the central advantages of iPSC-derived endothelial cells and SMC are their potential abundance by generating nearly unlimited numbers and their immune-privileged status as autologous tissue. Importantly, patient genomic information is maintained during the reprogramming and differentiation processes. Because iPSCs can be derived from subjects with genetic diseases, they represent an ideal vehicle to better understand a variety of diseases including heritable disorders, in particular the molecular mechanisms that dominate in vascular diseases, for screening of novel therapeutics and to foster vascular regeneration by cell replacement therapy or vascular grafts.

Although iPSCs start a new era of regeneration medicine, the tumorigenesis risk might compromise their further clinical applications. In turn, for vascular regeneration already robust selection markers and refined experimental protocols have been established to guide human iPSCs reproducibly to a vascular lineage. Additional negative selection against remaining pluripotent cells could be an additional option, to limit the risk of teratoma formation. Concerning the patient-derived autologous iPSC-differentiated cells, the respective genetic background is a benefit for vascular disease modeling studies; but because the same genetic or acquired abnormalities that predisposed a patient to a particular disease will be persist in their iPSCs might result in dysfunctional vascular cells or iPSC and resulting vascular progenies with reduced differentiation capabilities.

However, up to now, iPSC-derived vascular cells provide a reliable model system that enables basic vascular-biological research and molecular pharmacology studies with the potential to generate patient- specific disease models. In addition, the generation of clinically relevant numbers of vascular cells from human iPSC for use as a cell therapy in human subjects is already feasible.
